# The prognostic value of bispectral index and suppression ratio monitoring after out-of-hospital cardiac arrest: a prospective observational study

**DOI:** 10.1186/s13613-018-0380-z

**Published:** 2018-03-02

**Authors:** Ward Eertmans, Cornelia Genbrugge, Margot Vander Laenen, Willem Boer, Dieter Mesotten, Jo Dens, Frank Jans, Cathy De Deyne

**Affiliations:** 1 Department of Medicine and Life Sciences, Hasselt University, Diepenbeek, Belgium; 2 Department of Anaesthesiology, Intensive Care, Emergency Medicine and Pain Therapy, Ziekenhuis Oost-Limburg, Schiepse Bos 6, 3600 Genk, Belgium; 3 Department of Cardiology, Ziekenhuis Oost-Limburg, Schiepse Bos 6, 3600 Genk, Belgium

**Keywords:** Bispectral index, Suppression ratio, Prognostication, Out-of-hospital cardiac arrest, Neuromonitoring, Neurological outcome

## Abstract

**Background:**

We investigated the ability of bispectral index (BIS) monitoring to predict poor neurological outcome in out-of-hospital cardiac arrest (OHCA) patients fully treated according to guidelines.

**Results:**

In this prospective, observational study, 77 successfully resuscitated OHCA patients were enrolled in whom BIS, suppression ratio (SR) and electromyographic (EMG) values were continuously monitored during the first 36 h after the initiation of targeted temperature management at 33 °C. The Cerebral Performance Category (CPC) scale was used to define patients’ outcome at 180 days after OHCA (CPC 1–2: good–CPC 3–5: poor neurological outcome). Using mean BIS and SR values calculated per hour, receiver operator characteristics curves were constructed to determine the optimal time point and threshold to predict poor neurological outcome. At 180 days post-cardiac arrest, 39 patients (51%) had a poor neurological outcome. A mean BIS value ≤ 25 at hour 12 predicted poor neurological outcome with a sensitivity of 49% (95% CI 30–65%), a specificity of 97% (95% CI 85–100%) and false positive rate (FPR) of 6% (95% CI 0–29%) [AUC: 0.722 (0.570–0.875); *p *= 0.006]. A mean SR value ≥ 3 at hour 23 predicted poor neurological with a sensitivity of 74% (95% CI 56–87%), a specificity of 92% (95% CI 78–98%) and FPR of 11% (95% CI 3–29%) [AUC: 0.836 (0.717–0.955); *p *< 0.001]. No relationship was found between mean EMG and BIS < 25 (*R*^2^ = 0.004; *p *= 0.209).

**Conclusion:**

This study found that mean BIS ≤ 25 at hour 12 and mean SR ≥ 3 at hour 23 might be used to predict poor neurological outcome in an OHCA population with a presumed cardiac cause. Since no correlation was observed between EMG and BIS < 25, our calculated BIS threshold might assist with poor outcome prognostication following OHCA.

## Background

Once admitted to the Intensive Care Unit (ICU), post-anoxic brain injury is considered as the predominant cause of death in patients admitted after cardiac arrest (CA) [1, 2]. The implementation of targeted temperature management (TTM) in the post-CA setting improved neurological outcome substantially, but delayed neuroprognostication until at least 72 h after CA [3–7]. Nevertheless, early and reliable prognostication is most appreciated to inform relatives. Moreover, it could avoid futile and expensive treatment efforts in out-of-hospital cardiac arrest (OHCA) patients with irreversible brain damage.

Current guidelines recommend the use of a neuroprognostication algorithm including four main modalities which should be used in conjunction with each other whenever possible, i.e. clinical neurological examination, electrophysiology, biomarkers and brain imaging [5, 7, 8]. Unfortunately, most of the recommended prognostic markers are labour-intensive and above all require trained experts for correct interpretation. In recent years, the potential use of bispectral index (BIS) and to a lesser extent suppression ratio (SR) monitoring has been investigated in the post-CA setting [9–15]. Although these studies demonstrated that BIS and SR values could be used to predict poor neurological outcome, this monitoring option was not yet implemented in the neuroprognostication algorithm proposed by current guidelines [5]. Its use in brain-injured CA patients is namely associated with certain limitations. The BIS monitor was originally designed to monitor intraoperative awareness during anaesthesia, possibly implying that physicians treating post-CA patients are unfamiliar with its use [16]. Additionally, BIS monitoring is exposed to potential confounders of which high electromyographic (EMG) activity is acknowledged as the predominant one within CA research, causing falsely elevated BIS values [17]. In previous BIS studies, neuromuscular blockers (NMB) were administered continuously in all patients to minimize EMG activity interference although its constant use is not in line with current guidelines [18]. Altogether, these limitations question the clear benefit of this user-friendly monitoring option to assist with neuroprognostication. Therefore, this prospective, observational study aimed to assess the ability of BIS monitoring to predict poor neurological outcome in OHCA patients fully treated according to guidelines.

## Methods

### Study population

A prospective, observational study was performed between March 2011 and May 2015 in a Belgian tertiary care hospital (Ziekenhuis Oost-Limburg, Genk). All adult comatose survivors successfully resuscitated from OHCA with a presumed cardiac cause were admitted to the Coronary Care Unit (CCU) and were considered as eligible for this study. According to the institutional post-CA care protocol, all patients were treated uniformly and BIS monitoring was started immediately after admission to CCU [19]. Approval from the local Committee for Medical Ethics was obtained prior to study onset (11/06) and written informed consent was obtained from patient’s next of kin.

### Patient management

The institutional post-resuscitation protocol has been described previously [19, 20]. To summarize, TTM at 33 °C was initiated immediately after arrival at the emergency department by administering cold saline intravenously (4 °C—15–30 ml/kg). Urgent coronary angiography was performed followed by a percutaneous coronary intervention when indicated. TTM was further mechanically induced after CCU admission and maintained at 33 °C for 24 h using either endovascular (Icy cathether, CoolGard^®^ 3000, Alsius, Irvine, CA, USA) or surface (ArcticGel™ pads, Arctic Sun^®^ 5000, Medivance, Louisville, Colorado, USA) cooling systems. Subsequently, patients were rewarmed over the following 12 h (0.3 °C/h). Both cooling systems were equipped with a feedback loop system to control target temperature using an oesophageal temperature probe. All patients were intubated, mechanically ventilated and sedated with propofol, midazolam and remifentanil. Within the period of TTM, sedation doses were titrated to obtain values between − 3 and − 5 on the Richmond Agitation-Sedation scale. According to current guidelines, cisatracurium was only administered in case of shivering [18]. After the return to normothermia, sedation was reduced to evaluate patients’ neurological status properly. Patients not ready for extubation owing to circulatory or respiratory issues or due to persisting coma were kept sedated under the lowest dose needed to tolerate the endotracheal tube. EEGs were performed on clinical indication, and antiepileptic drugs were given in case of epileptic activity. Every EEG was characterized by a description of the posterior dominant rhythm (or absence thereof) and amplitude as well as the presence of non-dominant rhythms. Lateralization and the presence of artefacts was described where applicable. If present, epileptic activity was described as interictal, ictal or as status epilepticus. A status epilepticus was defined as continuous epileptic activity including rhythmic focal or generalized spikes lasting for more than 5 min and was considered as therapy-refractory in case of persistent epileptic activity in spite of at least two lines of antiepileptic drugs. Once the neurological, hemodynamic and respiratory status had been recovered sufficiently, patients were extubated.

### Bispectral index and suppression ratio monitoring

Bilateral BIS monitoring was initiated as soon as possible after CCU admission using the BIS VISTA™ (Aspect Medical Systems, Inc. Norwood, USA). A six-electrode frontotemporal bilateral sensor was applied to the patient’s forehead. The BIS monitor is a simplified EEG monitor that uses fast Fourier transformation to convert raw frontal EEG signals into simple and real-time BIS numbers ranging from 0 (iso-electric EEG) to 100 (normal electrical activity in awake subjects). Additionally, a SR is calculated, representing the percentage of each 63-s period that is iso-electric [21]. Finally, EMG activity is another parameter calculated by the BIS monitor describing the electromyographic content of the EEG signal and ranges from 0 (no EMG activity) to 100 (large EMG activity). BIS, SR and EMG values were continuously recorded and stored during the hypothermic and rewarming phase (i.e. 36 h). Although treating physicians (cardiologists) were not blinded to values displayed on the BIS monitor, decisions to withdraw life support were never based on these parameters and the dosage of sedatives was not titrated based on BIS values.

### Withdrawal of treatment policy

In patients not regaining consciousness despite complete cessation of sedation, maximal supportive treatment was provided until at least 72 h after normothermia was reached. Together with clinical neurological examination, malignant EEG patterns (i.e. status epilepticus, persisting burst suppression rhythms or long-lasting cerebral inactivity) were considered as the first-line support for the decision to withdraw therapy, and in specific, when seizures remained therapy-refractory or if other EEG rhythms with a poor prognosis remained present on subsequent EEG assessments. In case subsequent EEGs were inconclusive or when patients failed to wake up after the end of active temperature control, SSEPs and/or brain CT were performed. In line with international guidelines, absent corneal and pupillary reflexes, bilateral absence of the N20 component of SSEPs and refractory epileptic activity was considered to support this decision to withdraw therapy [22].

### Outcome assessment

The primary endpoint was neurological outcome defined by the Cerebral Performance Category (CPC) at 180 days post-CA. According to the scale classification, CPC1 is indicative for good cerebral performance; CPC2 implies moderate disability with sufficient cerebral functioning for independent daily-life activity; CPC3 indicates severe neurological sequelae; CPC4 implies coma or vegetative state; and CPC5 stands for death [23]. In this study, a CPC1–2 and CPC3–5 was considered as good and poor neurological outcome, respectively.

### Statistical analysis

Statistical analysis was performed using SPSS version 22.0 (SPSS Inc, Chicago, USA). Equal distribution was tested with a Kolmogorov–Smirnov test. Depending on normality, categorical data were compared between patients with a good and poor neurological outcomes using Fisher exact or Chi-Square tests, while unpaired t tests or Mann–Whitney *U* tests were used to compare continuous data. BIS, SR and EMG values were stored per second and left and right values were averaged. Mean BIS, SR and EMG values were then calculated per hour from initiation of TTM onwards and were used for data analysis. To assess the predictive ability of BIS and SR, receiver operating characteristic (ROC) curves were constructed at each hour using mean BIS and SR values and the Youden index was calculated for each ROC curve (Youden index = sensitivity + specificity – 1). The optimal time point and threshold to predict poor neurological outcome was determined based on the highest Youden index across all ROC curves. Relative risk ratios of poor neurological outcome were computed for the presence of single BIS and SR values between given intervals on the respective optimal time points. In addition, regression curves were fitted (including the calculation of the Pearson correlation coefficient) to describe the relationship between mean EMG and BIS values below and above our calculated (optimal) BIS threshold. Survival analyses were executed using Kaplan–Meier curves and log-rank statistic. *p* values < 0.05 were considered as significant.

## Results

Between March 2011 and May 2015, 121 eligible OHCA patients were consecutively enrolled in this study. Forty-four patients were excluded from further analysis for following reasons: no recording of BIS values (*n* = 34), initiation of BIS monitoring at day 2 (*n* = 4) and incoherence between time stamp and start of BIS monitoring (*n* = 6). In total, 77 successfully resuscitated OHCA survivors were prospectively included. At 180 days post-CA, 38 patients (49%) had a good (CPC1–2), while 39 patients (51%) had a poor neurological outcome (CPC5). There were no patients with a CPC 3 or 4 at 180 days following CA. Baseline characteristics, patient’s severity at admission and complications within the post-resuscitation management phase are summarized in Table 1 for both outcome groups. Sedation doses were in general higher in patients with a good neurological outcome. In total, 44 (57%) patients received NMB during the period of TTM, with no difference in the NMB dosage between both outcome groups (*p *= 0.804; Table 2).

**Table 1:** Baseline characteristics and post-resuscitation management and complications

Characteristic	Good neurological outcome (*N* = 38)	Poor neurological outcome (*N* = 39)	*p*
Age	67 ± 13	61 ± 13	*0.034*
Male	31 (82)	31 (80)	0.817
*Co-morbidities*			
Diabetes	2 (5)	10 (26)	*0.025*
Chronic kidney insufficiency	2 (5)	6 (15)	0.263
Cerebrovascular disease	2 (5)	2 (5)	1.000
Acute myocardial infarction	6 (16)	5 (13)	0.755
Arterial hypertension	16 (42)	17 (44)	1.000
Hyperlipidemia	15 (39)	16 (41)	1.000
*Cardiac arrest variables*			
Initial rhythm			*0.003*
Shockable	33 (87)	20 (56)	
Non-shockable	5 (13)	16 (44)	
Witnessed arrest	34 (92)	33 (87)	0.479
Bystander CPR	18 (47)	20 (51)	0.821
BLS duration (min)	8 (0–14)	10 (0–12)	0.561
ALS duration (min)	12 (8–21)	15 (10–28)	0.348
Number of shocks	2 (1–5)	1 (0–4)	0.111
Time emergency call—ROSC (min)	28 ± 19	32 ± 15	0.388
*Post-resuscitation management*			
Percutaneous Coronary Intervention	27 (71)	17 (44)	*0.015*
Cooling, endovascular/surface	20 (53)/18 (47)	15 (38)/24 (62)	0.265
Time to target temperature (min)	140 (73–295)	141 (107–195)	0.652
Intra-aortic balloon pump	11 (29)	6 (15)	0.178
*Post-resuscitation complications*			
Post-resuscitation shock	16 (42)	21 (54)	0.365
ARDS	4 (11)	7 (18)	0.517
Pneumonia	21 (55)	17 (44)	0.365
Acute kidney injury	9 (24)	12 (31)	0.610
Renal replacement therapy	3 (8)	3 (8)	1.000
Status epilepticus	1 (3)	20 (51)	< *0.001*
Burst suppression	4 (11)	16 (41)	*0.004*
*Cause of death*			
Neurological injury	–	27 (69)	–
Post-cardiac arrest shock	–	10 (26)	–
Other	–	2 (5)	–
CCU days	19 (12–32)	9 (6–17)	< *0.001*

Data are shown as mean ± SD, median with interquartile range and *n* (%)

*ALS* Advanced Life Support, *ARDS* Acute Respiratory Distress Syndrome, *BLS* Basic Life Support, *CCU* Coronary Care Unit, *CPR* cardiopulmonary resuscitation, *ROSC* return of spontaneous circulation

Statistical significant values indicate in italics (*p* < 0.05)

**Table 2:** Sedation doses and neuromuscular blockage

Sedatives	Good neurological outcome	Poor neurological outcome	*p* value
Propofol (mg/kg/h)	2.54 ± 0.51	1.35 ± 0.05	0.071
Remifentanil (µg/kg/min)	0.15 ± 0.07	0.10 ± 0.01	0.210
Midazolam (µg/kg/min)	1.45 ± 0.34	0.85 ± 0.21	0.156
Cisatracurium (mg/kg/h)^a^	0.13 (0.03–0.17)	0.10 (0.07–0.14)	0.804

Data are presented as mean ± SD and median with interquartile ranges

^a^Cisatracurium was administered in 20 and 24 patients with a good and poor neurological outcome, respectively

In 27 out of the 39 (69%) patients with a poor (neurological) outcome (CPC5), therapy was withdrawn at day 10 (6–20) post-CA and they died due to extensive neurological injury. First, 19 out of these 27 patients had a therapy-refractory status epilepticus. On top of persistent seizure activity, six and four out of these 19 patients had bilateral absent cortical responses on SSEP and diffuse brain oedema on CT, respectively. One patient with therapy-refractory seizures developed a septic shock after TTM at 33 °C ended and died 6 days after CCU admission. Another patient died 2 days after admission due to multi-organ failure in addition to persistent epileptic activity. Despite aggressive antiepileptic therapy, the other seven patients remained in a comatose vegetative state in whom therapy was withdrawn after 10 (9–32) days. Second, a bilateral absent cortical response (N20) on SSEP was the main reason for withdrawal of life-sustaining treatment in four out of these 27 patients. Third, two patients did not recover neurologically 2–3 weeks following CA, and EEGs persistently showed burst-suppression patterns. Finally, two patients had long-lasting cerebral inactivity based on EEG in whom a brain CT showed diffuse cerebral oedema indicative for extensive cerebral swelling.

**Fig. 1:**
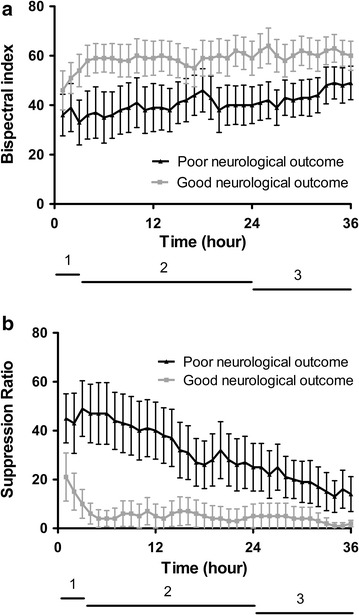
Evolution of mean BIS and SR during targeted temperature management. Hourly mean BIS (**a**) and SR values (**b**) are shown with their 95% CI in patients with a good and poor neurological outcome. Patients with a poor neurological outcome had significantly higher BIS and lower SR values during (1) the induction phase (*p *= 0.002 and *p *< 0.001, respectively), (2) the hypothermic phase (*p *< 0.001 and *p *< 0.001, respectively) and (3) rewarming phase (*p *< 0.001 and *p *< 0.001, respectively)

Figure 1 displays the evolution of mean BIS and SR values over the first 36 h from the initiation of TTM onwards in patients with good and poor neurological outcomes. After calculating the mean BIS and SR per hour, the optimal time point which provided the best sensitivity and specificity to predict poor neurological outcome was determined. A mean BIS value below or equal to 25 at hour 12 predicted poor neurological outcome with a sensitivity of 49% (95% CI 30–65%) and specificity of 97% (95% CI 85–100%) [AUC: 0.722 (0.570–0.875); *p *= 0.006]. Only one patient with a mean BIS ≤ 25 at hour 12 survived with a good neurological outcome. This corresponded to a false positive rate (FPR) of 6% (95% CI 0–29%). A mean SR value above or equal to 3 at hour 23 predicted poor neurological outcome with a sensitivity of 74% (95% CI 56–87%) and specificity of 92% (95% CI 78–98%) [AUC: 0.836 (0.717–0.955); *p *< 0.001]. This corresponded to a FPR of 11% (95% CI 3–29%). Three patients with a mean SR ≥ 3 at hour 23 had a good neurological outcome.

**Fig. 2:**
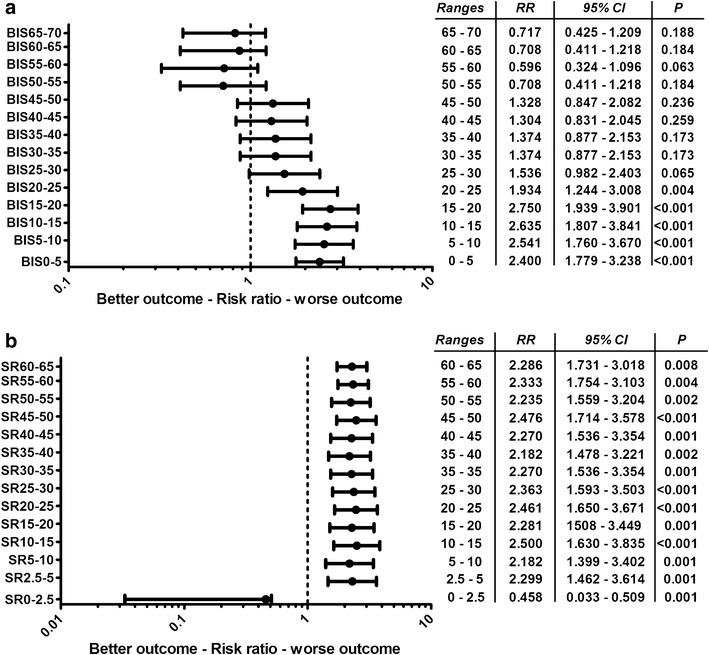
Forest plots. Relative risk ratios for poor neurological outcome at 180 days post-cardiac arrest are presented for the presence between given BIS (**a**) and SR (**b**) ranges at hour 12 and 23, respectively

At these optimal time points, relative risk ratios of poor neurological outcome were calculated for the presence of single BIS and SR values per second between given intervals (Fig. 2). Patients experiencing at least one BIS ≤ 25 at hour 12 had a 2.3-fold higher risk of poor neurological outcome (95% CI 1.38–3.85; *p *= 0.001). On the other hand, the presence of at least a single SR ≥ 3 at hour 23 was associated with a 4.4-fold higher risk of poor neurological outcome (95% CI 2.09–9.30; *p *< 0.001).

**Fig. 3:**
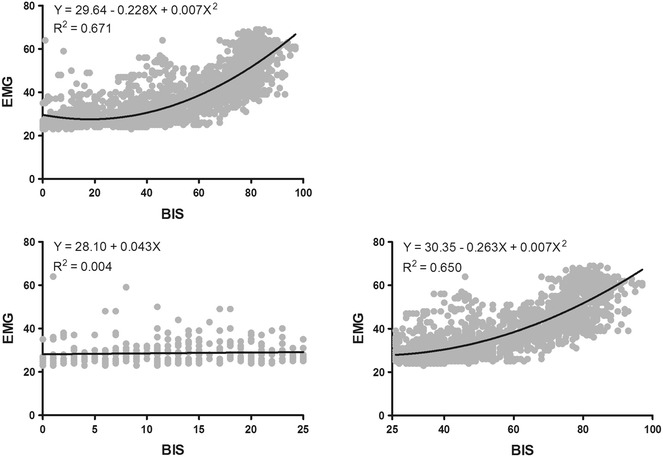
Correlation between EMG and BIS. The overall relationship between mean EMG and BIS is best described by a quadratic regression curve. No correlation is present between mean EMG and BIS below 25

The overall relationship between mean EMG and BIS was best described by a quadratic regression curve (*Y* = 29.64 − 0.228*X* + 0.007*X*^2^; *R*^2^ = 0.671; *p *< 0.001; Fig. 3). To account for possible EMG interferences on our calculated BIS threshold, regression curves were fitted between mean EMG and BIS values below and above 25. This analysis showed no relationship between mean EMG and BIS values below 25 (*Y* = 28.10 + 0.043*X*; *R*^2^ = 0.004; *p *= 0.209), implying that EMG interference below our calculated BIS threshold is rather negligible. In contrast, a significant relationship was observed between mean EMG and BIS values above 25 (*Y* = 30.35 − 0.263*X* + 0.007*X*^2^; *R*^2^ = 0.650; *p *< 0.001; Fig. 3).

**Fig. 4:**
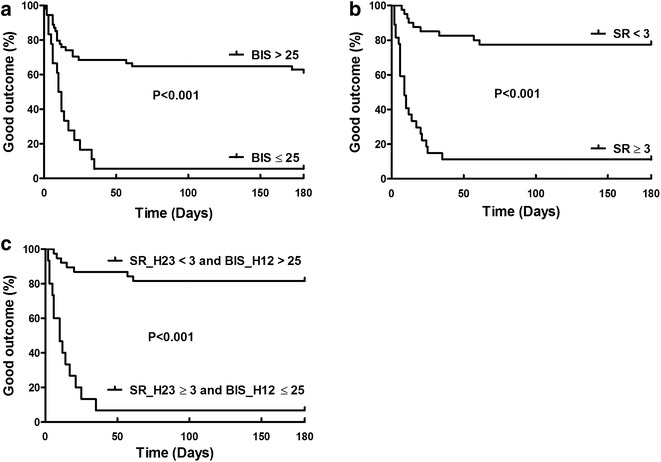
Survival analyses. Kaplan–Meier curves showing survival with a good neurological outcome according to BIS monitoring at hour 12 (**a**), SR monitoring at hour 23 (**b**) or both (**c**)

Survival curves are presented in Fig. 4. Patients with a mean BIS ≤ 25 at hour 12 were at high risk of poor neurological outcome (log-rank test *p *< 0.001; Fig. 4a). Patients with a mean SR ≥ 3 at hour 23 had a high risk for poor neurological outcome (log-rank test *p *< 0.001; Fig. 4b). A mean BIS ≤ 25 at hour 12 together with a mean SR ≥ 3 at hour 23 was associated with poor neurological outcome (log-rank test *p *< 0.001; Fig. 4c).

## Discussion

This study shows that BIS monitoring might assist with the prediction of poor neurological outcome in OHCA patients fully treated according to current guidelines. Mean BIS values below or equal to 25 at hour 12 and mean SR values above or equal to 3 at hour 23 were associated with poor neurological outcome in OHCA patients.

Consistent with previous studies, patients with a poor neurological outcome had lower BIS and higher SR values during the entire period of TTM. Up to now, the optimal time point and threshold to predict poor neurological outcome remain questionable. In the post-CA setting, mean BIS values below 40 or mean SR values above 40 during the first 4 h after cardiopulmonary resuscitation were shown to be early predictors for poor outcome [24]. According to Stammet et al. [14], a mean BIS value of 23 calculated over 12.5 h achieved a specificity and sensitivity of 89 and 86%, respectively. In line with these data, the optimal time point to predict poor neurological outcome in our patient cohort was at hour 12 using a mean BIS value of 25 reaching a specificity of 97%. As such, this is the first study which found a nearly similar BIS threshold to predict poor neurological outcome at a more or less identical time point. Furthermore, we demonstrated that a SR value of 3 at hour 23 had an even higher predictive power for poor outcome and its presence was associated with a fourfold higher risk to decease. Unfortunately, others did not assess the prognostic performance of SR longitudinally, which prevents us from comparing these results with current literature [10, 24]. For that reason, future studies are warranted to validate the value of SR as early prognostic marker in comparison to BIS.

BIS monitoring is known to be subjected to several confounding factors although this study confirms its potential to assist with early neuroprognostication [10, 11, 14, 15, 24, 25]. One of the predominant confounders is high EMG activity interference which is known to falsely elevate the BIS value [17]. In previous BIS studies, any influence of EMG activity on the BIS value was excluded by the continuous administration of NMB in all study patients although several undesirable effects have been associated with its routine use. Besides an increased risk on pneumonia and ICU-acquired weakness, continuous administration of NMB during TTM delays neurological examination and masks seizures [26–28]. Our patient cohort, however, was treated according to current guidelines, which suggest to limit the use of NMB to patients who experience shivering [18]. This allowed us to assess whether BIS monitoring was still able to predict poor neurological outcome after OHCA even though EMG activity interference was not minimized in all study patients. In fact, no correlation was observed between EMG and BIS values below or equal to our calculated threshold of 25, implying that EMG activity interference below this cut-off value is most likely negligible. Nonetheless, it is plausible to assume that the calculated sensitivity of our BIS threshold would have been higher if NMB were administered continuously.

In general, our results strengthen the hypothesis that BIS monitoring could be used as early prognostic tool after OHCA. Nevertheless, early neuroprognostication has always been challenging. It has become even more complicated within the era of TTM with guidelines currently suggesting to postpone prognostication until at least 72 h after OHCA [29, 30]. Still, it is desirable to identify patients with no prospect of full neurological recovery as early as possible. Therefore, prognostic markers should always achieve a high specificity with narrow confidence intervals. Using mean BIS values at hour 12 and mean SR values at hour 23, we were able to predict poor neurological outcome with a specificity of 97 and 92%, respectively. To compare our results, established neurophysiological tools such as SSEPs and EEGs reach a comparably high specificity [31, 32]. However, prognostic markers cannot be used on its own unless they reach a FPR of 0%. A false classification of patients with a favourable prognosis would namely result in an ethically unacceptable decision to withdraw medical treatment. Therefore, our results should be interpreted with caution and we do not advise to use BIS or SR as single parameter for outcome prediction. Currently, the benefit of a multimodal approach using an entire battery of prognostic markers is being investigated [12, 33]. Based on our results, future studies are needed to elucidate whether poor neurological outcome can be predicted with a false positive ratio of 0% by combining BIS and SR values with other highly specific parameters. For now, this study only confirmed the value of BIS and SR as potential early outcome predictors.

Several limitations need to be acknowledged. First, this was a single-centre study with a limited sample size. Second, physicians were not blinded since visual confirmation is required to assess signal quality. Nonetheless, BIS and SR values were not used in the decision of withdrawal of life-sustaining therapy. Third, it has been demonstrated that BIS values decrease under hypothermic conditions [34, 35]. Nevertheless, TTM at 33 °C unlikely influenced our results as all patients were treated uniformly and time to target temperature did not differ between both patient cohorts. Still, the ability of BIS monitoring to predict poor neurological outcome in patients treated with TTM at 36 °C remains to be elucidated. Finally, mean BIS and SR values were calculated per hour although these are not available in clinical practice. Nevertheless, Stammet et al. [14] demonstrated that a minute-by-minute analysis did not provide additional prognostic information. Therefore, we believe that mean BIS and SR values per hour should be implemented in the current BIS monitor as they might assist with neuroprognostication.

## Conclusions

This study shows that mean BIS values below or equal to 25 at hour 12 and mean SR values above or equal to 3 at hour 23 predicted poor neurological outcome in OHCA patients fully treated according to current guidelines. These results underline the possible potential of BIS monitoring to assist with early neuroprognostication in successfully resuscitated OHCA patients treated with TTM at 33 °C. Future studies are now warranted which should focus on the contribution of BIS and SR values to the multimodal neuroprognostication algorithm advised by current guidelines.

## Abbreviations

BIS: bispectral index
CA: cardiac arrest
CCU: Coronary Care Unit
CPC: Cerebral Performance Category
EEG: electro-encephalography
EMG: electromyographic activity
FPR: false positive rate
ICU: Intensive Care Unit
NMB: neuromuscular blockers
OHCA: out-of-hospital cardiac arrest
ROC: receiver operating characteristic
SR: suppression ratio
SSEP: somatosensory evoked potential
TTM: targeted temperature management
